# A Systematic Review of Antiaging Effects of 23 Traditional Chinese Medicines

**DOI:** 10.1155/2021/5591573

**Published:** 2021-05-15

**Authors:** Lixin Wang, Xu Zuo, Zhuoer Ouyang, Ping Qiao, Fang Wang

**Affiliations:** ^1^Department of Cell Biology, College of Basic Medical Sciences, Jilin University, Changchun 130021, China; ^2^Department of Pathogeny Biology, College of Basic Medical Sciences, Jilin University, Changchun 130021, China

## Abstract

**Background:**

Aging is an inevitable stage of body development. At the same time, aging is a major cause of cancer, cardiovascular disease, and neurodegenerative diseases. Chinese herbal medicine is a natural substance that can effectively delay aging and is expected to be developed as antiaging drugs in the future. *Aim of the review*. This paper reviews the antiaging effects of 23 traditional Chinese herbal medicines or their active components. *Materials and methods*. We reviewed the literature published in the last five years on Chinese herbal medicines or their active ingredients and their antiaging role obtained through the following databases: PubMed, EMBASE, Scopus, and Web of Science.

**Results:**

A total of 2485 papers were found, and 212 papers were screened after removing the duplicates and reading the titles. Twenty-three studies met the requirements of this review and were included. Among these studies, 13 articles used *Caenorhabditis elegans* as the animal model, and 10 articles used other animal models or cell lines.

**Conclusion:**

Chinese herbal medicines or their active components play an antiaging role by regulating genes related to aging through a variety of signaling pathways. Chinese herbal medicines are expected to be developed as antiaging drugs or used in the medical cosmetology industry.

## 1. Introduction

Aging is the degenerative change in the whole function of the organism that occurs with increasing age [1]. Aging is an extremely complex biological process, and its mechanisms involve the theory of genetic mutation, telomere loss, somatic mutation, free radical damage, immune disorder, mitochondrial dysfunction, and autophagy dysfunction [2, 3]. Cardiovascular disease, cancer, cataracts, osteoporosis, high blood pressure, and neurodegenerative diseases, such as Alzheimer's and Parkinson's diseases, are all linked to aging [4–6]. Given the aging population, many countries enter the aging society, which poses a serious threat to th economic development and human life and health. Therefore, the aging mechanism and antiaging drugs should be urgently studied [7].

The life cycle of the body involves a variety of signaling pathways and transcription factors, such as insulin and insulin-like (insulin/IGF-1 signaling [IIS]), dietary restriction (DR), gonad (germline signaling [GR]), and mitochondrial (mitochondrial signaling [mTOR]) signaling pathways [8–10]. These signaling pathways are shown to be conserved, which is a positive boost for the search for life-extending drugs and strategies to improve health [11, 12].

Studies have shown that the drug therapy can effectively delay aging and has a positive effect on aging-related diseases. Aspirin and metformin are commonly used synthetic antiaging drugs [13, 14], but these drugs also have significant side effects. Aspirin causes antiplatelet aggregation, and long-term use can easily cause bleeding, and patients taking metformin will have diarrhea, nausea, abdominal discomfort, and other adverse reactions [15, 16]. The discovery and development of antiaging drugs is difficult, and the progress is slow. Thus, finding a safe and effective antiaging drug is challenging. In recent years, Chinese herbal medicine has been considered a safe and effective antiaging drug with a great potential for development [17].

Herb-drug refers to the substance that is used to prevent and treat diseases and has the function of rehabilitation and health care under the guidance of Chinese medicine theory. [18]. Traditional Chinese medicine (TCM) is beneficial for chronic diseases [19]. For example, ginger has a significant effect on reducing circulating C-reactive protein (CRP) and tumor necrosis factor-alpha (TNF-*α*) levels, which are systemic inflammatory markers associated with an increased risk of cardiovascular disease [20]. Curcumin may ameliorate hyperandrogenemia and hyperglycemia associated with polycystic ovary syndrome [21]. Moreover, recent studies have found that a variety of TCM and their active components can delay aging and prevent age-related diseases [22, 23]. Polysaccharides, monopolysaccharides, or sesquiterpenes in TCM have anti-inflammatory, antitumor, antiviral, acaroid, and sedative effects, which are considered potential sources for the development of new drugs [24, 25]. In this paper, the antiaging and antioxidation effects of TCM or their active components are systematically reviewed.

## 2. Materials and Methods

The review was conducted following the Preferred Reporting Items for Systematic Reviews and Meta-Analyses (PRISMA) statement [26].

### 2.1. Search Strategy

English publications were searched from PubMed, Scopus, Embase, and Web of Science databases. All databases were limited to the Medical Subject Title Index (MESH/DECS) and available until 30 November 2020. Different combinations of the following keywords were used in the search: “traditional Chinese medicine”, “herbal medicine”, “TCM”, “aging”, “anti-aging”, “senescence” and “traditional medicine”. Besides, we looked at the references of all selected articles to find reports that were not found when we searched for articles.

### 2.2. Study Selection

By reading the titles and abstracts of the articles, the authors excluded the articles that did not meet the criteria of “Chinese medicine or its active ingredients to delay aging.” The antiaging effect of the TCM or its active components was studied in vitro and in vivo, and the possible mechanism was discussed. The following types of articles were excluded from consideration in this review: abstracts, editorial/letter review articles, meta-analyses, conference proceedings, case reports, patents, human studies, and articles published more than five years ago. The third author had the right to decide on any difference of opinion between the two authors.

### 2.3. Data Extraction

One author summarized the data in the paper, whereas the other examined the data. *Caenorhabditis elegans* is a classic model for the study of aging. A pair of data obtained from the experiment with *C. elegans* as a model is separately summarized in Table 1: types of TCM, role form, component analysis methods, main ingredients, dose, life expectancy increased, key genes, and pathways.  Table 2 summarizes the experimental data from other animal models or cell lines: types of TCM, role form action forms, animal models or cell lines, induced way, and pathways.

### 2.4. Methodological Quality Assessment

Optimized checklists were used to assess the risk and quality of bias of in vivo clinical studies [50, 51]. It includes blind administration, blind administration results, average treatment distribution, and other factors.

### 2.5. Data Analysis

Given the heterogeneity of the study, data were presented in narrative form, and no pooled statistics, sensitivity analysis, meta-analyses were used.

## 3. Results and Discussion

### 3.1. Search Results

**Figure 1:**
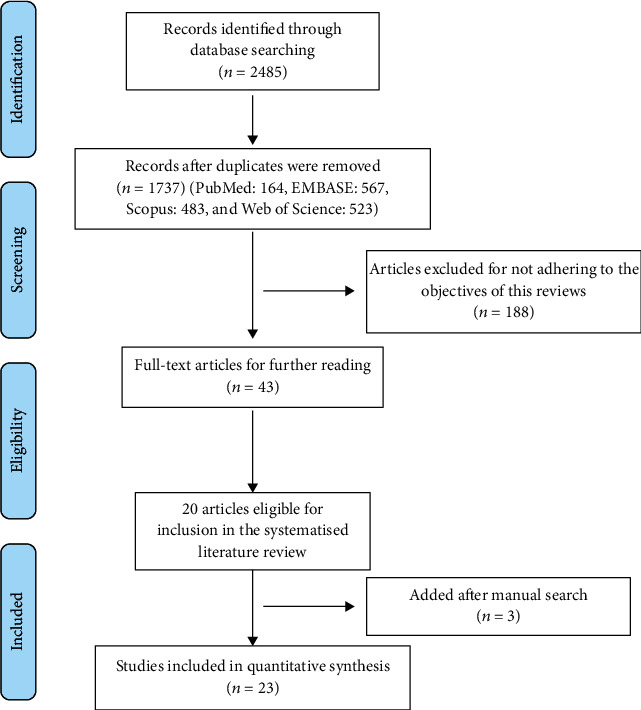
A flowchart of literature search and selection in this review is described in detail.

Figure 1 shows the search flowchart [52]. A total of 1737 articles are not duplicates (PubMed: 164, EMBASE: 567, Scopus: 483, Web of Science: 523). By reading the titles, we have selected 212 articles related to TCM and antiaging. Finally, through browsing the full text, 23 articles are recorded in this paper. Thirteen articles are based on *C. elegans* as animal models, and the other ten articles are based on other animals or cells.

### 3.2. Study Characteristics and Description

**Figure 2:**
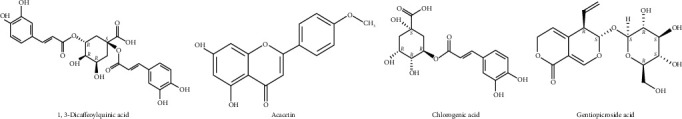
Chemical structures of some of the main components of TCM.

This paper presents a systematic review of 20 studies. Thirteen papers have used the classic *C. elegans* as an animal model to explore the effect of TCM or its main components on delaying aging. The other seven papers have studied the effects of TCM or its active components on silkworm, yeast, ultraviolet- (UV-) induced senescence cells, UV-induced skin senescence mice, and other aging models. A variety of TCM or their active components have evident inhibitory effects on aging. The chemical structure of some of the main components of Chinese medicine is shown in Figure 2. Moreover, 14 articles were from China; two, from India; two, from Germany; two, from Iran; three, from Korea; one, from Japan.

Several methods are reported in the antiaging experiments of TCM or its active components with *C. elegans* as the model. The longevity experiment is used to explore the effect of TCM on prolonging the lifespan of nematodes. The effects of TCM on the health status of nematodes are evaluated by measuring their body length and observing their body swing rate and locomotion ability. The effects of TCM on *C. elegans* resistance to stress are evaluated using heat, oxidative, and heavy metal stress tests. The fecundity of *C. elegans* is evaluated by counting the fecundity of nematodes and the sexual dominance rate of nematodes. The antioxidant capacity of TCM is evaluated by detecting the reactive oxygen species (ROS) and antioxidant enzyme levels in nematodes. The expression levels of various proteins and mRNAs are detected by transcriptome sequencing, Western blot, and Q-PCR. In addition, some articles have studied the effect of TCM on aging-related diseases.

In addition to the classic model of *C. elegans* to study the antiaging effect of TCM, yeast, silkworm, and other natural aging models are used in many experiments. Other aging models include hair dermal papilla (DP) cells, UV-induced hairless mouse skin aging model, UV-induced HaCaT cells and human dermal fibroblasts, H_2_O_2_-induced HUVECs aging, and D-galactose-induced aging mice. Methods include cell viability analysis, Western blot analysis, in situ staining for *β*-galactosidase activity, total collagen determination, stress tolerance and antioxidant activity, determination of antioxidant enzyme content, and other methods. Through the previously mentioned experimental methods, the evident antiaging effects of Chinese medicine or its active components are proven.

#### 3.2.1. Effect of TCM or Its Active Component on Prolonging the Life of *C. elegans*

*C. elegans* is a multicellular eukaryote that feeds on microorganisms. Nematodes have unique advantages, such as short life cycle, strong reproductive ability, highly homologous genes with mammals [53,54], and easy maintenance in experiments. These advantages have made them different from other animals. As a result, *C. elegans* has become the classic model of aging research.

The principal compounds in the clove essential oil (CEO) are caryophyllene, phenol, and 2-methoxyl-3-(2-propenyl) [27]. According to the US Food and Drug Administration, CEO is generally recognized as safe for use as food additive (U.S. Code of Federal Regulations, 21CFR184.1257). CEO significantly extends the nematode's lifespan and improves its reproductive capacity and health. CEO exerts its antioxidant activity by inducing the expression of *sod-3* and *gst-4*. In addition, CEO induces the *daf-16*/Forkhead box O (FOXO) nuclear transfer and induces germ cell apoptosis in a *cep-1* and d*af-16*-dependent manner.

Coix seed, a TCM with remarkable medical value, is widely planted in China and Japan. The coix seed oil (CSO) has blood lipid-lowering, antioxidation [55,56] and anticancer effects and can delay the aging of nematode worms [28]. Aging is closely related to environmental stress [57], but CSO can enhance nematode resistance to heat stress, oxidative stress, and heavy metal stress. CSO delays the aging of the nematode and enhances its stress resistance by inducing *daf-16* and its downstream genes. Linoleic, oleic, palmitic, and stearic acids in CSO play a key role in this process.

*Lonicera japonica* (LJ) is also known as Japanese honeysuckle [58], and its main component is chlorogenic acid [29]. Studies have shown that 75% ethanol extract of *L. japonica* (LJ-E) can prolong the life of nematodes through the insulin/IGF-1 signal transduction, antioxidant, and autophagy pathways. At the same time, LJ-E improves the health status of *C. elegans*, including the increase in the body swing and pharyngeal pumping frequencies, enhancement of resistance to heat and oxidative stress, and reduction in the ROS level in vivo. In addition, LJ-E and its extract can delay the aging of nematode and prevent Alzheimer's disease.

Glycyrrhizae radix (GR) is usually used in combination with other Chinese herbal medicines to treat peptic ulcers, hepatitis C, and skin diseases [59–62]. Recent studies have shown that the long-term exposure to GR can prolong the lifespan of nematodes, enhance their motor capacity, and reduce intestinal ROS production [30]. In addition, the GR treatment alters the expression pattern of genes encoding insulin-like signaling pathways, which play a key role in longevity control [11].

*Geng Nian Chun* (GNC) consists of 12 traditional Chinese medicines (i.e., *Radix Rehmanniae, Rhizoma Coptidis, Radix Paeoniae Alba, Rhizoma Anemarrhenae, Cistanche salsa,* and *Radix Morindae officinalis* [63]), which are used to improve functional loss associated with aging. The wild-type nematodes treated with GNC show prolonged survival time under normal and oxidative stress conditions, but the nematodes with *daf-16* mutation do not have antioxidant stress effects. This result suggests that the life extension and antioxidant stress effects of GNC are realized through the *daf-16*/FOXO-dependent pathway. Further study shows that GNC cannot prolong the lifespan of the mutant strains of *daf-2*, *age-1*, and *daf-16*. This result implies that GNC may extend the lifespan of nematodes through the IIS pathway and has a potential use in the development of antiaging drugs [31].

*Rehmannia glutinosa* (PRG), a TCM with remarkable medical value, has anti-inflammatory, antibacterial, and anticancer activities and can protect cardiovascular function. Recent studies have found that the main component of ripe PRG is a neutral polysaccharide. Among them, the neutral polysaccharide of NPRG, a functional pharmaceutical component, can regulate *daf-2* and *daf-16* genes through the IIS pathway, thereby enhancing the antistress ability and prolonging the lifespan of nematode worms [32].

*Lycium barbarum polysaccharide* (LBP) is one of the main active components of *L. barbarum*. LBP can prolong the lifespan of nematodes, improve their resistance to a harsh environment, enhance their reproductive ability, and ensure the integrity of nematode muscles. The RNAi gene is silenced with mutant nematode strains, and mRNA expression levels are measured. Using mutated nematode strains, RNAi silenced daf-16 genes of N2 and Sir-2.1 mutants, and measuring their mRNA expression levels, it was demonstrated that the life-prolonging activity of LBP is achieved by regulating *sir-2.1*, *daf-12*, and *daf-16* genes [33].

As a powerful antidote and immune system booster, juniper is often used to treat opportunistic infections [64]. Juniper essential oil (JBEO) extracted from juniper has certain antioxidant and anti-free-radical activities in vitro [65]. In addition, JBEO can prolong the lifespan of nematodes in vivo and improve the resistance of nematodes to oxidative stress and heat stress. Meanwhile, the increased expression of *sod-3* (39.49%) and *gst-4* (25.13%) is observed. In exploring the mechanism of JBEO life-prolonging activity, conserved transcription factors (i.e., *daf-16*, *skn-1*, and *hsf-1*) are found to be involved in this process [34].

Tambulin is a hydroxy iodic flavanol separated from *Zanthoxylum armatum*. Aging is a major cause of neurodegenerative diseases, including Huntington's syndrome, Parkinson's disease, and Alzheimer's disease [6,66]. The lifespan and stress tolerance of nematodes are significantly improved by tambulin treatment, and this phenomenon is accompanied by the remission of aging biomarkers, such as lipofuscin and protein carbonyl. Consistent with the decreased ROS level, the tambulin treatment results in the upregulated mRNA expression of ROS-removing genes, namely, *sod-1*, s*od-3*, and *ctl-2*. Tambulin therapy is shown to be effective in the treatment of Parkinson's disease; decreasing alpha-synuclein levels and lipid accumulation; improving motor behavior; elevating dopamine levels [35].

Previous studies have shown that *Hibiscus sabdariffa L.* can significantly reduce skin aging markers in female patients [67] and improve short- and long-term memory deficits in elderly albino mice [49]. According to recent studies that have used nematodes as animal models, *H. sabdariffa L.* extracts (HSE) can remarkably prolong the lifespan of nematodes in vivo and slow down the age-dependent decline in locomotor capacity [36]. This role of HSE depends on key transcription factors *daf-16* and *skn-1*. At the same time, HSE increases the intracellular ROS level, indicating that HSE has prooxidation activity. HSE is resistant to the toxicity induced by the amyloid-beta protein and has a life-prolonging effect.

*Polygonum multiflorum* extract (PME) can reduce the accumulation of lipofuscin in the liver and brain of mice [49] and has a neuroprotective effect on the degeneration of the substantia nigra striatum. Simultaneously, PME has an antioxidant effect, and nematodes exposed to PME have enhanced antioxidant stress ability. In addition, PME can prolong the average lifespan of *C. elegans* and reduce the accumulation of ROS by regulating *daf-16* and *sir-2.1* [37].

At present, the clinical application of *Ganoderma lucidum* is limited to adjuvant therapy, such as regulating immune response and reducing inflammatory response [68,69], but its pharmacological mechanism remains unclear. Recent studies have shown that *G. lucidum* can effectively improve the resistance of nematodes to paraquat-induced oxidative stress and heavy metal stress and can extend their lifespan. The protective effect of *G. lucidum* on nematodes may be exerted through dietary restriction and the mTOR/S6K signaling pathway, whereas the lifespan extension of nematodes is dependent on the germline signaling pathway [38].

*Astragalus* armor glycoside IV (AS-IV) is isolated from dry *Astragalus* root and is widely used in the treatment of inflammation, viruses, and even cancer [70]. The lifespan of AS-IV-treated nematodes is prolonged under oxidative stress, heat stress, and normal conditions. At the same time, AS-IV can enhance the activities of superoxide dismutase (SOD) and peroxidase, increase the content of glutamic acid, and decrease the content of glucose in nematodes. Interestingly, the lifespans of *sod-1*, s*od-2*, *sod-3*, *sod-4*, *sod-5*, *ctl-1*, *ctl-2*, *ctl-3*, and *daf-16* mutants do not change with AS-IV treatment. These results indicate that the life-prolonging activity of AS-IV is achieved by improving the age-related functional decline and antioxidant capacity and partially regulating the activity of the IIS pathway [39].

#### 3.2.2. Effect of TCM or Its Active Components on Delaying Senescence in Other Animal Models or Cells

Aside from the *C. elegans* model, silkworm, yeast, and other natural aging models with short life cycle are used in antiaging research of TCM or its active ingredients. In addition to the natural aging model, UV-induced senescence cells and hairless mice are commonly used to simulate skin aging; DP cells aging model was used to simulate hair loss; H_2_O_2_-induced HUVECs aging was used to study cardiovascular diseases. In addition, D-galactose-induced aging mice are one of the main means of antiaging drug research. Chinese medicines or their active components have an antiaging effect in the body and a significant inhibitory effect on skin aging.

*Snutellaria baicalensis* Georgi flowers extract (SFE) is mainly composed of flavonoids that can improve spatial memory ability. Studies have shown that SFE can significantly regulate malondialdehyde (MDA), SOD, and advanced glycation end products and significantly improve liver pathological abnormalities. In addition, SFE significantly increases the levels of D-glutamine and D-glutamate. SFE is speculated to play an antiaging role by regulating the glutamine–glutamate metabolism pathway [40].

The water extract of *Rhodiola Rosea* can significantly extend the lifespan of silkworms and enhance their resistance to heat stress and hunger without changing their food intake, body weight, or fertility. At the same time, *R. rosea* treatment increases the activities of glutathione S-transferase and catalase and changes the contents of glutathione and MDA. In addition, the mRNA expression of BmFOXO is significantly increased after *R. rosea* treatment [41]. BmFOXO is a key transcription factor in the IIS pathway and acts downstream of the IIS pathway [71]. Therefore, the IIS may be involved in the prolonged silkworm life induced by *R. rosea*.

*Gentiopicroside* (GPS), which is isolated from *Gentiana rigescens,* is an iridoid glycoside compound with an antiaging effect. GPS can effectively prolong the replication and chronological lifespan of yeast, improve the survival rate of yeast under oxidative stress, and enhance the activities of catalase, SOD, and glutathione peroxidase. In addition, the levels of free GFP in the cytoplasm, free GFP in the mitochondria, and ubiquitin are significantly increased after GPS treatment. Autophagy, especially mitochondrial autophagy, and antioxidant stress may be involved in the GPS-induced life extension [42].

The aqueous extract (WEZ) and volatile oil (VOZ) of *Zanthoxylum bungeanum* Maxim can alleviate memory impairment and protect against D-galactose-induced hippocampal nerve injury. In addition, WEZ and VOZ enhance the activity of phosphatidylinositol 3-kinase (PI3K)/proteinase B (Akt). The evident therapeutic effect of *Z. bungeanum* on memory disorders may be related to the activation of the PI3K/Akt signaling pathway [43].

Male mice are induced to senescence after the subcutaneous injection of D-galactose for 42 days. Treatment with *Nigella sativa* fixed oil reduces the lipid peroxidation in mice. *N. sativa* fixed oil (0.1 and 0.2 mL/kg) significantly restores the GSH content and reduces Bax/Bcl2 levels. In addition, 0.1 mL/kg *N. sativa* fixed oil downregulates the expression of caspase-3 protein in the brain and liver of aging mice. *N. sativa* fixed oil may play an antiaging role in D-galactose-induced aging models through its antioxidant activity and antiapoptotic effects [44].

HaCaT cells and human dermal fibroblasts (HDF) are induced by UV light, and cells are aged. Safflower seed oil (*Charthamus tintorius* L., SSO) and its main component acacetin (5,7-dihydroxy-4′-methoxyflavone) inhibit the expression of matrix metalloproteinases (MMP-1) in aging HaCaT and HDF cells [72]. MMP-1 plays an important role in collagen degradation and wrinkle formation. SSO and acacetin may inhibit skin aging through MMP-1 [45].

UV radiation can produce ROS that damages the skin structure and causes skin aging. Skin aging can be simulated through the UV irradiation of hairless mice [73]. The treatment of pomegranate juice concentrated powder (PCP) can significantly improve skin wrinkling and edema caused by photoaging and significantly increase the content of skin moisture, type I collagen, and hyaluronic acid. In addition. glutathione consumption is inhibited by PCP therapy. Moreover, PCP decreases the expression levels of MMP-1, 9, and 13 and NOX2 mRNAs in the skin of mice exposed to UV. PCP has a good protective effect on skin aging induced by UVB [46].

*Agastache rugosa Kuntze*, a perennial herb, belongs to the mint family (Lamiaceae). *A. rugosa* has been shown to contain several kinds of flavonoids, including acacetin-7-O-*β*-D-glucopyranoside (tilianin), acacetin, linarin, agastachoside, and rosmarinic acid [74]. Hot water extract of *Agastache rugosa* Kuntze leaf (ARE) can attenuate the UV-B-induced ROS generation and reduce the activity and protein level of ProMMP-2 and -9 induced by UV-B and increase the activity level of total GSH and total SOD reduced by UV-B in HaCaT keratinocytes [47]. The protective effect of ARE on UV-B-induced photoaging in HaCaT keratinocytes may be based on the upregulation of antioxidant components, including total GSH and SOD.

DP cells play an important role in the occurrence and development of androgenetic alopecia (AGA) [75]. Aging DP cells may participate in the occurrence of AGA by upregulating the expression of SRD5A2. *Plumbago zeylanica* (also known as Chitrak) and its components can promote the growth of DP cells and downregulate the expression of SRD5A2 in DP cells [48]. It is speculated that *Plumbago zeylanica* may be used to treat AGA.

Endothelial cell aging is a major risk factor for inducing cardiovascular disease (CVD) [76, 77]. The vascular endothelial dysfunction induced by hydrogen peroxide (H_2_O_2_) is partly responsible for the development of aging [78, 79]. Ginsenoside RB1 (RB1) is the main component of ginsenoside, which has biological activities such as relieving oxidative stress, antiobesity, and anti-inflammation [80–82]. Studies have shown that RB1 restored the H_2_O_2_-induced reduction in SIRT1 expression and activated AMPK phosphorylation to protect HuVecs from H_2_O_2_-induced senescence [81]. This provides a new way to prevent cardiovascular diseases associated with aging.

### 3.3. Methodological Quality/Risk of Bias

**Figure 3:**
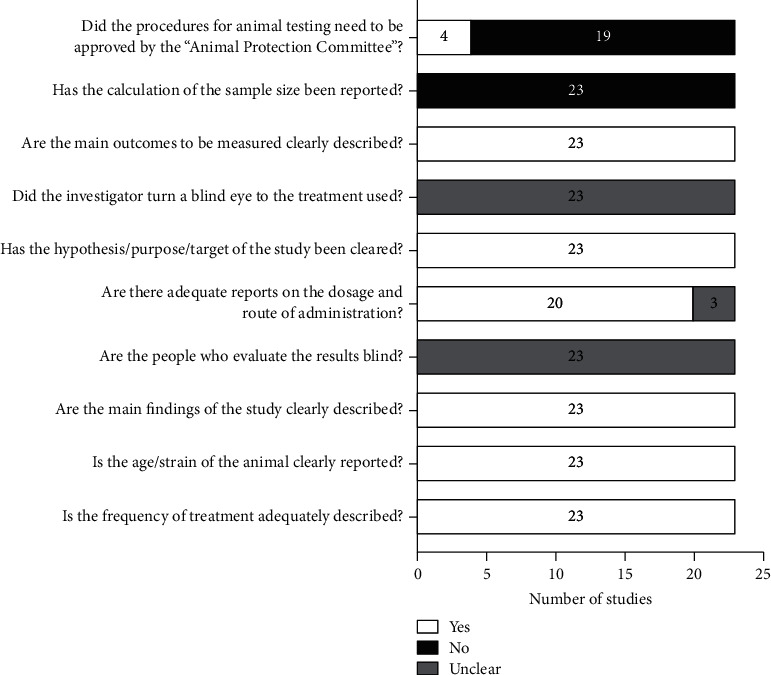
Methodological quality of included in vivo studies.

Figure 3 introduces the methodological features of this review. In all papers, the frequency of TCM treatment and age and strain of experimental animals are described in detail. Experiments using *C. elegans* and silkworm as animal models do not need the approval of the animal protection evaluation committee, and experiments requiring support have already been approved. The main purpose and findings of the study have been accurately expressed in all articles.

**Figure 4:**
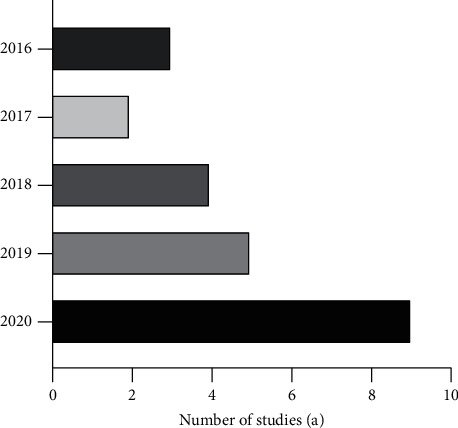
The year of publication of the review article.

**Figure 5:**
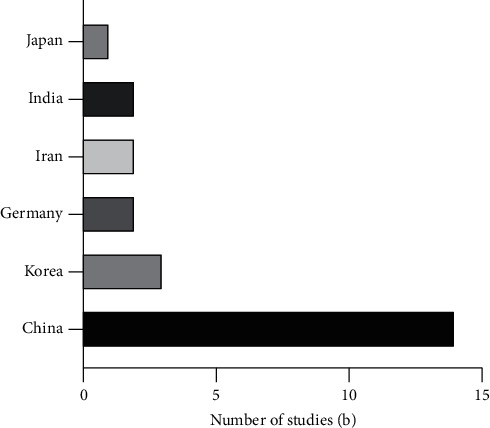
The country of publication of the review article.

Figures 4 and 5 present the year and country of publication of each article in the review. From the perspective of expression years, the number of articles published is increasing yearly. As a traditional treatment method, TCM is gaining new vitality, and its antiaging effect is also attracting increasing attention. In terms of publishing countries, Chinese herbal medicine is widely studied in China, Japan, South Korea, Germany, India, and Iran. Chinese herbal medicine has been widely valued worldwide, and its in-depth research has promoted the development of new drugs based on the natural products of plants.

## 4. Conclusion

This paper reviews the antiaging and antioxidant potentials of TCM or its active components as natural products. TCM or its active components play a significant antiaging effect in various aging models. Considering the rigor of this review, although improvement is still needed in some aspects, the quality of the articles included in the review is of a medium or high level.

The IIS pathway is the first confirmed pathway to regulate aging [83]. From humans to nematodes, this longevity control pathway has always been highly conserved [84, 85]. The IIS pathway acts through the PI3K/Akt and is activated by insulin peptides. *Age-1* and *daf-2* encode phosphoinositol-3 kinase (PI3K) and insulin/IGF-1 receptors [86, 87], respectively, which are key upstream components of IIS. Decreased *daf-2* function leads to inactivation of downstream kinase cascades beginning with AGE-1/PI3K [88]. Downregulation of *age-1* inactivates 3-phosphoinositol-dependent kinase 1 (PDK-1) [89]. This, in turn, downregulates the Akt/protein kinase B (PKB) family members, AKT-1 and AKT-2 [89]. The PI (3, 4, 5) P 3/PI (4, 5) P 2 ratio can also be decreased by the activation of DAF-18/phosphatase and tensin (PTEN) phosphatase, which mediates dephosphorylation of PI (3, 4, 5) P3 and increases lifespan [88, 90–92]. FOXO/DAF-16 plays an important role in the PI3K/Akt pathway. Under weak insulin signaling conditions, unphosphorylated FOXO/DAF-16 is transported to the nucleus to promote the transcription of genes related to longevity in the organism [93, 94]. Through a systematic review of 20 studies, we have found that most of the antiaging effects of TCM are involved in the IIS pathway. Coix seed essential oil, clove essential oil, *Lonicera japonica* crude extractions, Glycyrrhizae Radix crude extractions, Gengnianchun aqueous extractions, *Rehmannia glutinosa* neutral polysaccharides, *Lycium barbarum* neutral polysaccharides, juniper berry (*Juniperus communis* L.) essential oil, *Zanthoxyllum aramatum* natural flavonol, *Hibiscus sabdariffa* L., *Polygonum multiflorum* aqueous extract, and *Astragalus* membranaceus astragaloside IV (AS-IV) can all increase the expression of *daf-16* [22–32,34]. *Hsf-1* expression in *C. elegans* was increased after treatment with coix seed essential oil and *Lonicera japonica* crude extractions [23,24]. After treatment with *Rehmannia glutinosa* neutral polysaccharides, *Lycium barbarum* neutral polysaccharides, and *Lonicera japonica* crude extractions, the expression pattern of *daf-2* in *C. elegans* was low [24, 27, 28].

Sirtuin family is a kind of nicotinamide dinucleotide (NAD+) dependent deacylase, which plays a significant role in preventing diseases and delaying senility [95, 96]. Levels of sirtuins, including silencing information regulator 1 (SIRT1) and silencing information regulator 6 (SIRT6) but not silencing information regulator 2 (SIRT2), have been reported to decline in senescent cells exposed to oxidants in mouse embryonic fibroblasts, lung epithelial cells, human endothelial cells, and macrophages [97, 98]. Sirtuin plays an active role in maintaining gene integrity [96], regulating telomere reverse transcriptase expression [99], promoting DNA repair [100–102], changing the expression of senescence related genes, and maintaining stem cell self-renewal [103,104]. Sirtuin also regulates body longevity. The life span of the budding yeast *Saccharomyces cerevisiae*, nematodes, *Drosophila melanogaster*, and mice would be prolonged with the increase of sirtuin levels [105–108]. Sirtuins were found to interact with all major longevity conserved pathways, such as AMP-activated protein kinase (AMPK), insulin/IGF-1 signaling pathway (IIS), target of rapamycin (TOR), and forkhead box O (FOXO) [109–111]. It was found that the expression level of sir-2.1 gene was increased after LBP treatment, and the longevity prolonging effect of LBP on the sir-2.1 mutant was shorter than that of N2. This implies that the life-extending effect of LBP requires sir-2.1 [33]. In addition, *Polygonum multiflorum* can prolong the life span of wild-type *Caenorhabditis elegans* and improve its ability to resist paraquat stress, but not the SIR-2.1-deficient strain [37]. Curcumin pretreatment significantly reduced H_2_O_2_-induced premature senescence of HUVECs, which was characterized by decreased percentage of senescence associated *β*-galactosidase positive cells, enhanced cell division ability, and decreased expression of senescence associated protein p21 [112]. SIRT1 short interfering RNA (siRNA) inhibition of SIRT1 can reduce the expression and phosphorylation of eNOS and eliminate the protective effect of curcumin on H_2_O_2_-induced premature senescence. These results suggested that curcumin could reduce the premature senility of HUVECs induced by oxidative stress by activating SIRT1. It has been found that ginsenoside can reduce the positive rate of *β*-galactosidase in H_2_O_2_-induced HUVEC. In addition, RB1 reduced eNOS acetylation and promoted more NO production, accompanied by an increase in SIRT1 expression. Interestingly, after SIRT1 was knocked out, the effect of RB1 on HUVEC aging was weakened [113].

The continuous research on the antiaging effect of TCM and the exploration of the antiaging pathway will be helpful in the research and development of new antiaging drugs.

## Figures and Tables

**Table 1:** Antiaging research of TCM and its active components with *Caenorhabditis elegans* as a model.

Types of TCM	Role form	Component analysis method	Main ingredients	Dose	Life expectancy increased	Key genes	Pathways	References
Clove	Essential oil	Gas chromatography–mass spectrometry (GC–MS)	Aryophyllene; phenol; 2-methoxyl-3-(2-propenyl)	1 mg/ml	15.3%	*daf-16; sod-3*; *gst-4; cep-1*	Antioxidant pathway; insulin/IGF-1 signaling pathway (IIS); apoptosis pathway	[27]
Coix seed	Essential oil	GC–MS	Linoleic acid; oleic acid; palmitic acid	1mg/ml	22.79%	*mev-1*; *hsf-1*; *daf-16*	Antioxidant pathway	[28]
*Lonicera japonica*	Crude extractions	High performance liquid chromatography (HPLC)	Chlorogenic acid; 1,5-dicaffeoylquinic acid; 1,3-dicaffeoylquinic acid	500 *μ*g/ml	21.87%	*mev-1*; *hsf-1*; *daf-16*; *daf-2*; *sod-3*	Antioxidant pathway; IIS	[29]
Glycyrrhizae radix	Crude extractions	Reverse phase high-performance liquid chromatography (RP-HPLC)	Liquiritin; isoliquiritin; glycyrrhizic acid	0.24 g/ml	—	*daf-16*; *daf-18*; *pdk-1*	IIS	[30]
Gengnianchun	Aqueous extract	—	—	3.94 mg/ml	31.3%	*age-1*; *daf-16*	IIS	[31]
*Rehmannia glutinosa*	Neutral Polysaccharides	UPLC analysis; FR-IR spectrum	Galactose, glucose, and arabinose	—	—	*sod-3*; *daf-16*; *daf-2*	Antioxidant pathway; IIS	[32]
*Lycium barbarum*	Neutral Polysaccharides	Phenol-sulfuric acid method; HPLC-GPC; FR-IR spectrum; GC-MS	Mannose, glucose, and galactose	300 *μ*g/ml	20.72%	*daf-16*; *daf-2*; *daf-12*; *sir-2.1*	IIS	[33]
Juniper berry (*Juniperus communis* L.)	Essential oil	GC-MS	*α*-pinene; limonene	10 ppm	18.5%	*sod-3*; *gst-4: daf-16*; *skn-1*	Antioxidant pathway; IIS	[34]
*Zanthoxyllum aramatum*	Natural flavonol; tambulin	—	—	50 *μ*M	16.79%	*sod-1*; *sod-3*; *stl-2; daf-16*	Antioxidant pathway; IIS	[35]
*Hibiscus sabdariffa* L.		—	—	1mg/ml	24%	*daf-16*; *skn-1*	—	[36]
*Polygonum multiflorum*	Aqueous extract	—	—	1000 *μ*g/ml	18.6%	*daf-16*; *sir-2.1*	IIS	[37]
*Ganoderma lucidum*	Aqueous extract	—	—	7.5 mg/ml	—	*eat-2; rsks-1*	mTor/s6k pathway; dietary restriction pathway	[38]
*Astragalus membranaceus*	Astragaloside IV (AS-IV)	—	—	—	27.8%	*sod-3*; *sod-4*; *sod-5*; *daf-16*	Antioxidant pathway; IIS	[39]

**Table 2:** Antiaging research of TCM or its active components based on other animal or cell models.

Types of TCM	Role form	Animal models or cell lines	Induced way	Pathways	Ref.
*Scutellaria baicalensis* Georgi flowers	—	Rat	D-galactose-induced	Glutamine–glutamate metabolic pathway	[40]
*Rhodiola rosea*	Aqueous extract	Silkworm; *Bombyx mori*	—	IIS	[41]
*Gentiana rigescens* Franch	Gentiopicroside (GPS)	Yeast	—	Mitochondrial autophagy pathway; antioxidant pathway	[42]
*Zanthoxylum bungeanum* Maxim (Rutaceae)	Aqueous extract, volatile oil (VOZ), petroleum ether (PEZ), and methylene chloride	Mice	D-galactose-induced	PI3K/Akt/Nrf2 signaling pathway	[43]
*Nigella sativa*	Fixed oil	Mice	D-galactose-induced	Antioxidant pathway; antiapoptotic pathway	[44]
Safflower seed	Oil	HaCaT cells and HDF	Ultraviolet B-induced	—	[45]
Pomegranate	Dried pomegranate juice	Mice	UVB-induced	—	[46]
*Agastache rugosa* Kuntze	Hot water extraction	HaCaT	UVB-induced	—	[47]
*Plumbago zeylanica*	—	Dermal papilla cells	—	—	[48]
Ginseng	Ginsenoside Rb1	Human umbilical vein endothelial cells (HUVEC)	H_2_O_2_-induced	SIRT signaling	[49]
